# Brain Signature Characterizing the Body-Brain-Mind Axis of Transsexuals

**DOI:** 10.1371/journal.pone.0070808

**Published:** 2013-07-26

**Authors:** Hsiao-Lun Ku, Chia-Shu Lin, Hsiang-Tai Chao, Pei-Chi Tu, Cheng-Ta Li, Chou-Ming Cheng, Tung-Ping Su, Ying-Chiao Lee, Jen-Chuen Hsieh

**Affiliations:** 1 Department of Psychiatry, Taipei Veterans General Hospital, Taipei, Taiwan; 2 Integrated Brain Research Unit, Department of Medical Research and Education, Taipei Veterans General Hospital, Taipei, Taiwan; 3 Department of Psychiatry, Shuang Ho Hospital, Taipei Medical University, Taipei, Taiwan; 4 Department of Dentistry, School of Dentistry, National Yang-Ming University, Taipei, Taiwan; 5 Department of Obstetrics and Gynecology, Taipei Veterans General Hospital, Taipei, Taiwan; 6 Department of Obstetrics and Gynecology, Faculty of Medicine, School of Medicine, National Yang-Ming University, Taipei, Taiwan; 7 Department of Medical Research and Education, Taipei Veterans General Hospital, Taipei, Taiwan; 8 Department of Psychiatry, Faculty of Medicine, School of Medicine, National Yang-Ming University, Taipei, Taiwan; 9 Institute of Brain Science, National Yang-Ming University, Taipei, Taiwan; 10 Center for Neuropsychiatric Research, National Health Research Institutes, Zhunan, Taiwan; Catholic University of Sacred Heart of Rome, Italy

## Abstract

Individuals with gender identity disorder (GID), who are commonly referred to as transsexuals (TXs), are afflicted by negative psychosocial stressors. Central to the psychological complex of TXs is the conviction of belonging to the opposite sex. Neuroanatomical and functional brain imaging studies have demonstrated that the GID is associated with brain alterations. In this study, we found that TXs identify, when viewing male-female couples in erotic or non-erotic (“neutral”) interactions, with the couple member of the desired gender in both situations. By means of functional magnetic resonance imaging, we found that the TXs, as opposed to controls (CONs), displayed an increased functional connectivity between the ventral tegmental area, which is associated with dimorphic genital representation, and anterior cingulate cortex subregions, which play a key role in social exclusion, conflict monitoring and punishment adjustment. The neural connectivity pattern suggests a brain signature of the psychosocial distress for the gender-sex incongruity of TXs.

## Introduction

On September 14, 2011, the Australian government declared that Australian citizens could register their preferred gender identities on their passports, with medical certificates from their regular physicians, to travel without fear of discrimination [1]. Furthermore, in May of 2012, the Argentinian government passed the Gender Identity Law that warrants free choice of gender [2]. These reforms were revolutionary steps for the evolution of human rights with regard to gender identification, signifying a shift away from negative social stereotypes. In the Diagnostic and Statistical Manual of Mental Disorders, Fourth Edition, Text Revision (DSM-IV-TR), gender identity disorder (GID) is characterized as a strong and persistent cross-sex identification and discomfort with the original sex or a sense of inappropriateness in the role of that sex [3], [4]. In this report, we studied individuals with GID, commonly referred to as transsexuals (TXs). Throughout their lives, TXs consistently suffer from negative psychosocial stressors [5]–[8].

At the behavioral level, TXs may focus on their identification with their desired genders under both erotic and regular circumstances. At the level of neural processing, there may be neural correlates that are functionally associated with the experience of psychosocial distress related to the gender-sex incongruity. Therefore, we performed a functional magnetic resonance imaging (fMRI) study to investigate the functional connectivity between the midline [9] structures of the brain and the ventral tegmental area (VTA). Among the midline structures, in particular, we imaged the anterior cingulate cortex (ACC), a neural structure central to the consciousness of self and conflict monitoring as well as social processing [9], [10]. The ventral tegmental area (VTA) is a neural substrate that is involved in dimorphic genital representation and the rewarding value of sexual behaviors [11], [12].

In the ACC, we specifically probed the regions engaged in processing social exclusion [13], [14], emotional conflicts in interpersonal relationships [15] and behavioral adjustments for punishment [16], i.e., the dorsal ACC (dACC) and pregenual ACC (pgACC). Furthermore, previous studies have suggested that the resting-state functional connectivity in low-frequency oscillations, as studied by fMRI, may reflect the brain state of the subject's self-referential internal representation [17], exteroceptive/interoceptive focus of attention [18] and the readiness of the brain to engineer an instant mind operation [19]. Such functional links, which are sculpted by sustained associative learning, can act as a form of “system memory” that recapitulates the history of experience-driven co-activation of cortical circuitries [20].

## Materials and Methods

### Participants and clinical assessment

Individuals who met the diagnostic criteria for GID according to the DSM-IV-TR were recruited from the psychiatric clinic of Taipei Veterans General Hospital (TXs, n = 41, refusal rate for participating in the experiment of 41%). Heterosexual age-matched normal controls (CONs, n = 38, 19 males and 19 females) were recruited from electronic bulletin board systems via the internet.None of the participants in the study had undergone sex reassignment surgery, and they did not have any other neurological or major psychiatric disorders (see Table 1 for a complete list of the exclusion and inclusion criteria for participant selection). The TXs consisted of 2 subgroups: 21 female-to-male TXs (FTMs) and 20 male-to-female TXs (MTFs). MTFs are biological males who identify themselves as and have the desire to be females, whereas FTMs are biological females who identify themselves as and have the desire to be males. The TXs were either hormone-treated (H^+^) or untreated (H^−^). H^+^/MTFs received estrogen or anti-androgen, whereas H^+^/FTMs received testosterone for hormone therapy. The TXs underwent regular follow-ups and were assessed and diagnosed using psychiatric diagnostic interviews according to the DSM-IV-TR [3]. For brain connectivity studies, 23 H^−^/TXs and 23 age-matched CONs (fc/CONs) from the behavioral study participated in fMRI analysis (Table 2). Females and FTMs were not ovulating or pregnant during the study period, and participants abstained from sexual behavior the day prior to the experiment.

**Table 1:** Inclusion and exclusion criteria for the TX group and the CON group.

*Inclusion criteria for the CON group*	
1.	Written informed consent approved by the institutional review board (IRB)
2.	Aged 20–40 years old
3.	Sexual orientation according to the Klein Sexual Orientation Grid: Average score of A to G<4 and total scores <56 (heterosexual sexual orientation)
*Inclusion criteria for the TX group*	
1.	After a clinical psychiatric interview according to the Diagnostic and Statistical Manual of Mental Disorders (DSM-IV-TR), transsexuals met the criteria for GID and no other major psychiatric disorders. They had not received sexual reassignment surgery.
2.	Written informed consent approved by the institutional review board (IRB)
3.	Aged 20–40 years old
4.	Sexual orientation according to the Klein Sexual Orientation Grid: Average score of A to G>4 and total scores >56 (homosexual sexual orientation)
*Exclusion criteria (for both the TX and the CON groups)*	
1.	Visual problems (except those corrected by glasses)
2.	Current or previous physical or neurological diseases
3.	Current medical treatments
4.	Did not meet the diagnosis of other psychiatric disorders (except for GID in the TX group)
5.	Arizona Sexual Experience Scale (ASES) total score >18 or score of any item >5
6.	With experience watching erotic films and those with feelings of disgust most of the time (more than half) during their experiences watching erotic films.
7.	A history of sexual abuse
8.	Females in their ovulatory period*
9.	Sexual contact leading to orgasm 24 hours before the study
10.	Consuming alcohol, tea or coffee 24 hours before the study
11.	Pregnancy
12.	Not applicable for MRI study

*Subjects who were in the period ranging from less than 11 days (follicular phase) or more than 17 days after the beginning of their last menses were included. Follow-up phone calls were made to verify the date of the beginning of the next menses. This selection criterion was used on the basis that sudden surges in LH (luteinizing hormone) and FSH (follicle stimulating hormone) at mid-menstrual cycle could affect brain activation patterns.

**Table 2:** Demographic data and the results of psychological assessments.

	Behavioral study						Neuroimaging study		
	CONs	TXs	P value	H^+^/TXs	H^−^/TXs	*P* value	fc/CONs	H^−^/TXs	P value
	n = 38	n = 41		n = 18	n = 23		n = 23	n = 23	
**Age**	25.2±4.2	26.5±5.7	0.51^a^	27.8±6.5	25.4±5.0	0.34^a^	24.4±4.6	25.4±5.0	0.51^a^
**Education**	15.7±1.6	14.6±2.4	0.04* a	14.8±2.6	14.5±2.3	0.56^a^	15.9±1.1	14.5±2.3	0.02* a
**Marriage**			1.0^b^			0.19^b^			
** single**	38(100.0%)	39(95.1%)		16(88.9%)	23(100.0%)		23(100.0%)	23(100.0%)	
** married**	0(0.0%)	1(2.4%)		1(5.6%)	0(0.0%)		0(0.0%)	0(0.0%)	
** divorced**	0(0.0%)	1(2.4%)		1(5.6%)	0(0.0%)		0(0.0%)	0(0.0%)	
**Employment**			0.67^b^			0.86^b^			0.23^b^
** employed**	19(50.0%)	24(58.5%)		10(55.6%)	14(60.9%)		8(34.8%)	14(60.9%)	
** unemployed**	2(5.3%)	1(2.4%)		0(0.0%)	1(4.3%)		2(8.7%)	1(4.3%)	
** student**	17(44.7%)	16(39.0%)		8(44.4%)	8(34.8%)		13(56.5%)	8(34.8%)	
**Sexual orientation**			<0.001*** b			0.16^b^			<0.001*** b
** homosexual**	0(0.0%)	33(80.5%)		14(77.8%)	19(82.6%)		0(0.0%)	19(82.6%)	
** heterosexual**	38(100.0%)	3(7.3%)		1(5.6%)	2(8.7%)		23(100.0%)	2(8.7%)	
** bisexual**	0(0.0%)	2(4.9%)		0(0.0%)	2(8.7%)		0(0.0%)	2(8.7%)	
** none**	0(0.0%)	3(7.3%)		3(16.7%)	0(0.0%		0(0.0%)	0(0.0%)	
**Psychological assessments**									
** IOS**	1.0±1.6	9.5±0.8	<0.001*** a	9.7±0.7	9.2±0.8	0.02* a	1.0±1.7	9.2±0.8	<0.001*** a
** DOS**	1.0±1.7	9.6±0.9	<0.001*** a	9.7±0.8	9.5±1.0	0.26^a^	1.1±1.6	9.5±1.0	<0.001*** a
** BDI**	4.9±3.8	14.2±11.7	<0.001*** a	9.9±9.1	17.6±12.7	0.06^a^	3.8±3.0	17.6±12.7	<0.001*** a
** DSQ**									
** undoing**	4.0±1.3	4.9±1.7	0.02* a	4.4±1.4	5.2±1.8	0.25^a^	3.9±1.5	5.2±1.8	0.02* a
** altruism**	5.9±1.3	6.6±1.7	0.02* a	6.3±1.9	6.8±1.5	0.34^a^	5.9±1.5	6.8±1.5	0.04* a
** displacement**	3.4±1.1	4.4±1.6	0.003** a	4.5±1.4	4.3±1.8	0.53^a^	3.3±1.1	4.3±1.8	0.03* a

***Behavioral study.*** Demographic data and the scores of psychological assessments were compared between TXs and CONs (total participants) and between hormone-treated (H^+^/TXs) and untreated (H^−^/TXs) subjects.

***Neuroimaging study.*** Demographic data and the scores of psychological assessments were compared between H^−^/TXs and fc/CONs for subjects participating in the neuroimaging experiment. Age and education are presented as the mean ± standard deviation in years. The percentage denotes the proportion of participants with a particular status within the group. Only sub-categories of the DSQ that reached statistical significance are listed.

a The two-tailed Mann-Whitney *U*-test was used for between-group comparisons of continuous variables.

b Fisher's exact test was used for between-group categorical variables. The asterisks indicate the level of significance.

*P<0.05;

**P<0.01;

***P<0.001.

All participants' sexual orientation was assessed using clinical interviews, medical histories and the Klein Sexual Orientation Grid (Table 1). All participants were evaluated regarding the level of their *self-identification as the opposite sex* (IOS, “*Please rate the degree to which you identify yourself as another gender opposite to your biological sex*”) and the *desire to become the opposite sex* (DOS, “*Please rate the degree to which you desire to become another gender opposite to your biological sex*”) using a visual analog scale (0 =  none and 10 =  the maximum imaginable). The TXs, compared to the CONs, reported very high levels of IOS and DOS (Table 2). The psychosocial defenses of the participants were assessed using a validated Chinese version of the Defense Style Questionnaire (DSQ) [21], [22], and their moods were assessed using the Beck Depression Index (BDI) (Table 1) [23]. The study was approved by the institutional review board of Taipei Veterans General Hospital, and written consent was provided by all participants.

### Behavioral study

The participants watched 4 silent erotic (E) and 4 silent neutral (N) films (30-sec duration each) in a balanced semi-randomized order. These films (maximized for arousal and minimized for disgust in the E-films; minimized for eliciting emotion in the N-films) were randomly chosen from a database of films that were previously validated with other groups of TXs and CONs. The E-films contained scenes of male-female genital intercourse in the nude, whereas the N-films contained scenes of common male-female dialogue in regular clothing. For the psychological ratings, participants used a visual analog scale (0 =  none and 10 =  the maximum imaginable). Immediately after each film, the participants rated their erotic arousal (*arousal* score) according to the statement “*Please rate the degree to which you felt sexually aroused when watching this film*” and the extent to which they felt embodied as male/female (*selfness* score) according to the statement “*Please rate the degree to which you identify yourself as the male/female in the film*.” For this study, we were mainly interested in comparing the TXs as one group (MTFs and FTMs combined) to the CONs as one group (heterosexual males and females combined), which was also justified by the fact that neither MTFs and FTMs nor female CONs and male CONs differed in age, education, IOS, or DOS (data not shown). To discern a possible hormone-treatment confound in the combined groups, we also compared H^+^/TXs and H^−^/TXs in regard to behavioral variables.

### fMRI experiment

We performed a functional connectivity analysis of the fMRI data (fcMRI) during the resting state. Only H^−^/TXs participated in the neuroimaging study to avoid the possible confounding effects of gonadal hormone treatment on brain activity [24]. This subgroup retained all of the features of the larger TX group (Table 2). Resting-state fMRI images were obtained using a 3.0-T MRI scanner (Discovery MR750, GE Inc., USA) at the Taipei Veterans General Hospital. The scanning parameters were as follows: gradient echo T2* weighted sequence, [TR]/[TE]  = 2,000 ms/30 ms, [FOV]  = 230×230 mm^2^, matrix size  = 64×64, 40 slices/image volume, slice thickness  = 4 mm and 205 volumes per run. The initial 5 scans were discarded for signal saturation. During scanning, the participants were instructed to remain awake with their eyes open and fixated on a cross sign on the projection screen. Participants were instructed to remain relaxed. High-resolution T1 structural images were acquired in the sagittal plane using a high-resolution sequence (TR/TE = 8.208 ms/3.248 ms, FOV = 230×230×158.4 mm^3^, matrix  = 256×256×176).

### Statistical analysis

#### Behavioral study

Averaged *arousal* and *selfness scores* were first obtained for the E- and N-films for each individual. Within-group comparisons of *arousal* and *selfness* ratings for the desired genders (i.e., the gender that a TX cross-identifies with) and un-desired genders (i.e., the gender that a TX feels discomfort with, or the original sex) while watching the E- and N-films were then conducted using the Wilcoxon signed-rank test. For between-group comparisons (CONs vs. TXs, H^+^/TXs vs. H^−^/TXs and H^−^/TXs vs. fc/CONs), the Mann-Whitney *U*-test was employed.

#### fcMRI analysis

Preprocessing was carried out using an advanced edition of the Data Processing Assistant for Resting-State fMRI (DPARSF) (http://www.restfmri.net) [25], which is based on Statistical Parametric Mapping (SPM8) (http://www.fil.ion.ucl.ac.uk/spm) and the Resting-State fMRI Data Analysis Toolkit (REST, http://www.restfmri.net) [26]. All scans were slice timing-corrected, head movement-corrected and normalized to the Montreal Neurological Institute (MNI) template. Spatial smoothing was performed with a 6-mm full-width half-maximum Gaussian kernel. The low-frequency time series was extracted with a band-pass filter (0.009 Hz<f<0.08 Hz), and spurious or nonspecific sources of variance were removed by regression of the following variables: (a) the 6 movement parameters computed by rigid body translation and rotation in preprocessing, (b) the mean whole brain signal, (c) the mean signal within the lateral ventricles and (d) the mean signal within the deep white matter.

VTA-seeded voxel-based correlation analysis was then performed by anchoring the VTA as the seed region (defined as a sphere with a 3-mm radius centered at the MNI coordinates [4, −18, −12]) [11]. For each participant, a correlation map disclosing the correlation of the time series between the VTA and each voxel was created. One-sample *t*-tests (P*_uncorrected_*  = 0.001, k = 20) were performed on the individual correlation maps to create group connectivity maps respectively for H^−^/TXs and fc/CONs. To examine the hypothesis that TXs may show altered functional connectivity between the VTA and the dACC/pgACC, VTA-seeded region-of-interest (ROI, radius  = 10 mm)-based analysis with a small volume correction (SVC, controlled for the family-wise error, P = 0.05) was performed on the connectivity map respectively for H^−^/TXs and fc/CONs. The a priori ROIs specifically relate to the processing of social exclusion [13], [14], emotional conflict in interpersonal relationships [15] and behavioral adjustments in response to punishment [16]. Intergroup comparisons (H^−^/TXs vs. fc/CONs) were performed using similar procedures (threshold set at P*_uncorrected_*  = 0.005, followed by a more conservative SVC procedure, corrected for family-wise error, P = 0.05).

## Results

### Behavioral study

**Figure 1:**
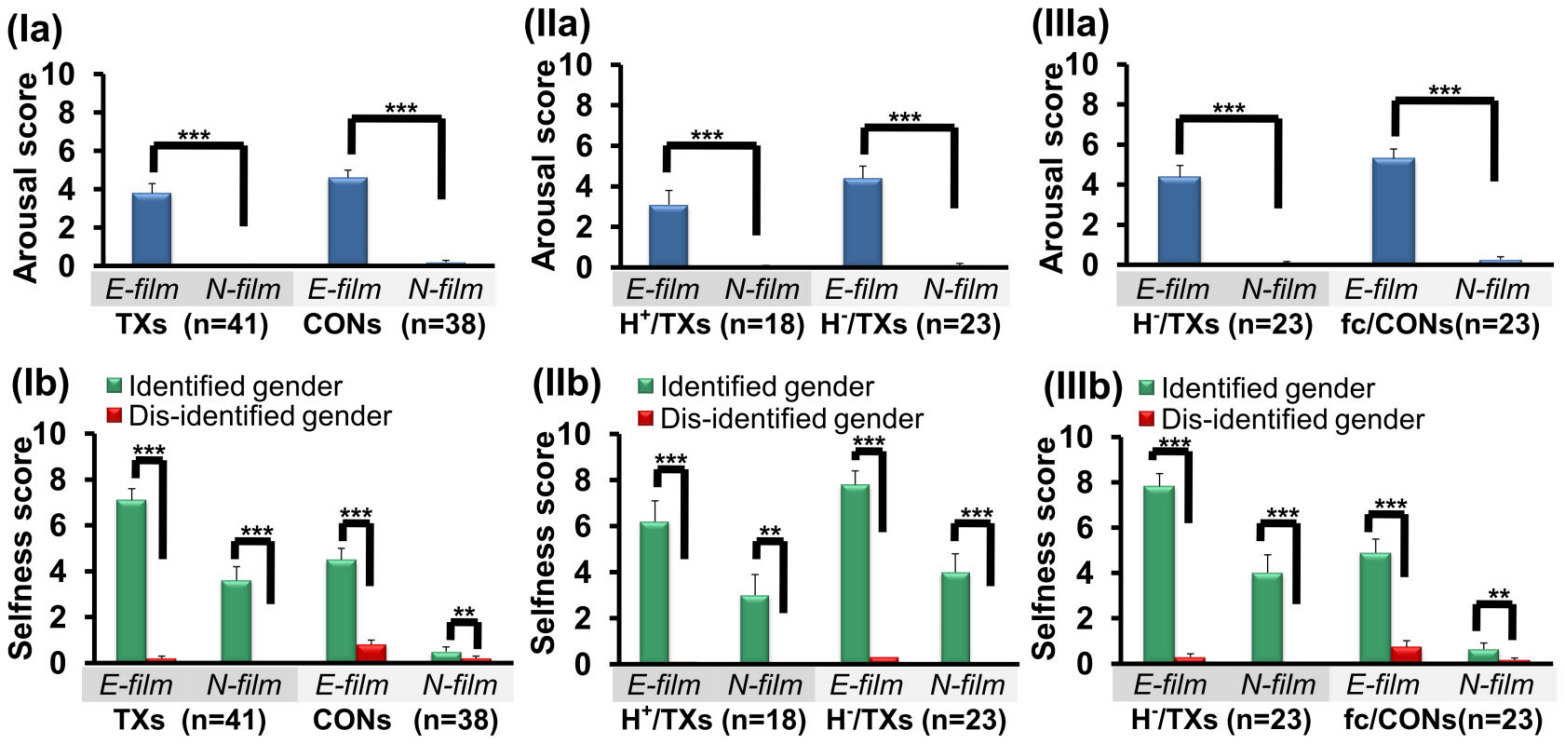
Behavioral and neuroimaging data. *Panel Ia. Arousal score*. Both TXs and CONs were highly aroused when watching the E-films. *Panel Ib. Selfness score*. Both TXs and CONs demonstrated high selfness ratings for their desired genders as opposed to the un-desired genders while watching the E-films. TXs, compared to CONs, rated higher *selfness* scores (P<0.001) for their desired genders when watching the E-films. Notably, TXs also reported high selfness ratings even while watching the N-films. In contrast, the CONs did not show such significant differences compared to the TXs and featured much lower *selfness* scores for their desireed genders when watching the N-films (P<0.001). *Panel IIa. Arousal score*. Both hormone-treated (H^+^/TXs) and untreated (H^−^/TXs) subgroups rated significantly higher levels of *arousal* when watching the E-films vs. N-films. *Panel IIb. Selfness score*. Both hormone-treated (H^+^/TXs) and untreated (H^−^/TXs) subgroups rated significantly higher *selfness* scores for the desired gender compared to the un-desired gender when watching the E-films and N-films. *Panel IIIa* and *Panel IIIb*. The imaging subgroups (H^−^/TXs and fc/CONs) showed similar rating patterns as those of their original groups (TXs and CONs, respectively). The two-tailed Wilcoxon signed-rank test was used for within-group comparisons. The asterisks indicate the level of significance. ** P<0.01, *** P<0.001.

All groups rated higher levels of *arousal* for the E-films compared to the N-films (TXs, P<0.001; CONs, P<0.001), and all groups rated higher levels of *selfness* (TXs, P<0.001; CONs, P<0.001) when they identified themselves as the desired genders vs. the un-desired genders while watching the E-films. Compared to the CONs, TXs rated higher *selfness* scores (P<0.001) for their desired gender when watching the E-films. Notably, TXs demonstrated significantly higher *selfness* scores (P<0.001) when they saw themselves as their desired gender vs. un-desired gender while watching the N-films. In contrast, the CONs did not show such significant differences when compared to the TXs for the N-films (Fig. 1, panel Ia and Ib), and the CONs reported much lower *selfness* scores for the desired genders when watching the N-films (P<0.001). Further analysis of the H^+^/TXs and H^−^/TXs subgroups revealed that both subgroups had similar psychological profiles and conformed to the results of the TX group as a whole (Fig. 1, panel IIa and IIb). Finally, the behavioral profiles from the comparisons between H^−^/TXs vs. fc/CONs were similar to those for the comparisons between TXs and CONs (Fig. 1, panel IIIa and IIIb). These findings confirm that the imaging subgroups reflected the same psychological features as the original groups.

### fcMRI study

**Figure 2:**
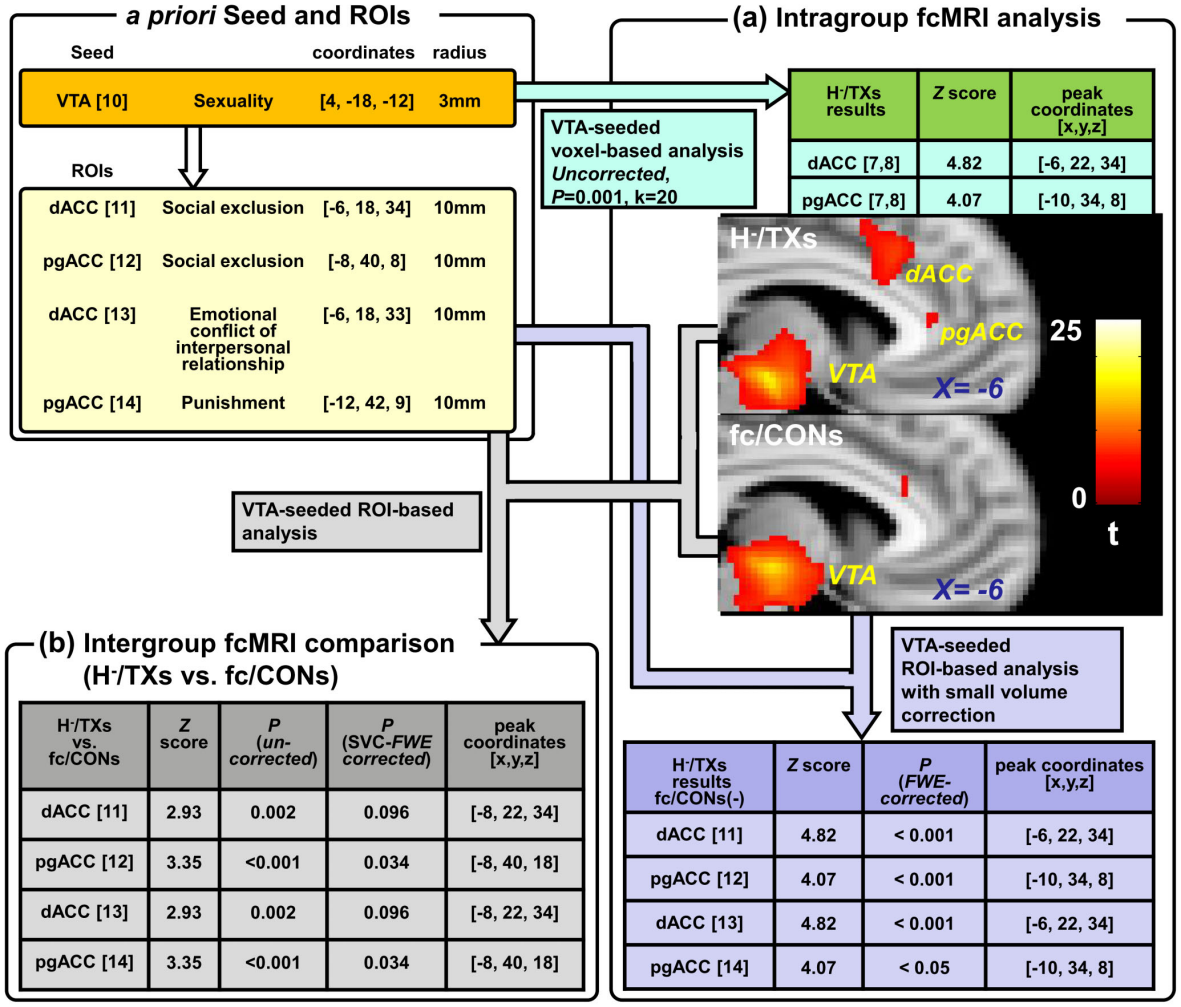
A priori voxel-based and ROI-based correlation analyses. *Panel a. Intragroup comparison*. Voxel-based correlation analysis was performed by anchoring the VTA as the seed region (defined as a sphere with a 3-mm radius centered at the MNI coordinates [4, −18, −12]) [11]. The a priori ROIs specifically relate to the processing of social exclusion [13], [14], emotional conflict in interpersonal relationships [15] and behavioral adjustments in response to punishment [16]. VTA-seeded ROI (radius  = 10 mm)-based analysis with a small-volume correction(SVC, controlled for the family-wise error, P = 0.05) was performed based on the connectivity map for H^−^/TXs and fc/CONs. *Panel b. Intergroup comparison*. The same VTA-seeded ROI-based analysis was performed using the contrast map (H^−^/TXs >fc/CONs) with and without a SVC. [X] indicates the cited reference.

In the VTA-seeded voxel-based correlation analysis, the dACC (P*_uncorrected_* <0.001, *Z* = 4.82, peak coordinates  = [−6, 22, 34]) and the pgACC (P*_uncorrected_* <0.001, *Z* = 4.07, peak coordinates  = [−10, 34, 8]) values showed significant positive connectivity with the VTA only among the H^−^/TXs (Fig. 2, panel a). In the VTA-seeded ROI-based analysis, only H^−^/TXs demonstrated a positive correlation with the VTA and the specific subregions of the dACC and pgACC that are involved in the processing of social exclusion, emotional conflicts and punishment (P*_corrected_* <0.05 for all ROIs). Note that the peak coordinates determined by the ROI analyses were co-localized with the peak coordinates within the dACC and pgACC clusters disclosed by the VTA-seeded correlation analysis (Fig. 2, panel a). For intergroup comparison between H^−^/TXs and fc/CONs, the same a priori VTA-seeded ROI-based analysis revealed that the selected ROIs demonstrated a significantly greater positive correlation with the VTA (P*_uncorrected_*  = 0.002 for dACC and <0.001 for pgACC). In particular, the pgACC survived the more conservative SVC procedure (P*_corrected_* <0.05) (Fig. 2, panel b).

## Discussion

There exist neurobiological factors that underpin the spectrum of gender identity. Recent anatomical and neuroimaging studies have revealed an association of transsexualism with functional and structural changes of the brain [27]–[31]. In addition, the pattern of brain activity in TXs has been demonstrated to substantially overlap with that of their desired genders when they encounter gender-related stimuli, such as visual erotic stimuli or odorous steroids [32], [33].

Anatomically, it has been reported that a female-sized bed nucleus of the stria terminalis was found in the MTFs [34]. An additional study showed that the volume and neuron number of the interstitional nucleus of the anterior hypothalamus, INAH3, of MTFs were similar to those of control females, and that those of one FTM fell within the range of control males [27]. It has also been demonstrated that transsexualism can be associated with a different cerebral gray-matter pattern from that of controls [28]. These findings suggest that brain anatomy may play a role in gender identity [27], [28], [31]. Diffusion tensor imaging studies have also revealed that the white matter microstructural patterns in untreated FTMs are closer to the patterns of subjects who share their gender identity than those who share their biological sex [29], [30]. These findings suggest that TXs and non-TX controls differ in neuroanatomical feature. Furthermore, it has been reported that MTFs may be associated with sex-atypical neuronal responses in specific hypothalamic circuits during odorous stimulation of gonadal hormones with pheromone-like properties, possibly as a consequence of variant neuronal differentiation [32]. MTFs may manifest brain activation pattern overlapping that of normal females during the viewing of erotic stimuli [33]. Collectively, these findings indicate that GID is characterized by structural and functional alterations in the brain.

Central to the psychological complex of TXs is the conviction of belonging to the opposite sex, i.e., a conspicuous incongruence between their desired psychological gender and un-desired biological sex. This fact was evidenced by the very high ratings of IOS and DOS given by TXs in the clinical assessments (Table 2). Behaviorally, TXs demonstrated high saliency of their desired gender in both erotic and neutral conditions while CONs did so only in erotic situation. Furthermore, psychosocial maladaptation in TXs was evidenced by the maladaptive defensive styles found in the DSQ.

It is noteworthy that the behavioral results from TXs may be influenced by a *self-presentation bias*; TXs with this bias may tend to exaggerate their cross-gender features to justify their cross-gender needs. For example, these individuals may exaggerate the desire to be another gender to receive a positive opinion from the psychiatrist (for further medical or surgical treatment). We meticulously and explicitly instructed the TXs that the experiments of this study were intended only for pure scientific research, as the results were in no way of any relevance or influence to their clinical diagnosis and future medical intervention.

The VTA, which has a pivotal role in dopamine-mediated reward circuitry, is a neural substrate that holds the dimorphic genital representation and the rewarding of sexual behaviors [11], [12]. The dACC and the pgACC have been associated with conflict monitoring/error processing and mentalizing/self-referential social processing [9], [10], and both regions targeted in this study, dACC and pgACC, are engaged in processing experiences of social exclusion [13], [14]. The dACC is also engaged when experiencing emotional conflict during interpersonal interaction [15], whereas the pgACC participates in behavioral adjustment to punishment [16]. In the context of “system memory”, defined as the neural representation of sustained associative learning [20], the fcMRI-disclosed functional association of the VTA, dACC and pgACC may represent a central representation of the psychosocial distress related to the gender-sex incongruency of TXs.
